# Parallel and Practical Approach of Efficient Image Chaotic Encryption Based on Message Passing Interface (MPI)

**DOI:** 10.3390/e24040566

**Published:** 2022-04-18

**Authors:** Mohammed Abutaha, Islam Amar, Salman AlQahtani

**Affiliations:** 1 College of Information Technology and Computer Engineering, Palestine Polytechnic University, Hebron P.O. Box 198, Palestine; 161100@ppu.edu.ps; 2 New Emerging Technologies and 5G Networks and Beyond, Computer Engineering Department, College of Computer and Information Sciences, King Saud University, Riyadh 11451, Saudi Arabia; salmanq@ksu.edu.sa

**Keywords:** MPI, parallel encryption, chaotic system, IoT, 5G, cybersecurity

## Abstract

Encrypting pictures quickly and securely is required to secure image transmission over the internet and local networks. This may be accomplished by employing a chaotic scheme with ideal properties such as unpredictability and non-periodicity. However, practically every modern-day system is a real-time system, for which time is a critical aspect for achieving the availability of the encrypted picture at the proper moment. From there, we must improve encryption’s performance and efficiency. For these goals, we adopted the distributed parallel programming model, namely, the message passing interface (MPI), in this study. Using the message passing interface, we created a novel parallel crypto-system. The suggested approach outperforms other models by 1.5 times. The suggested parallel encryption technique is applicable.

## 1. Introduction

The fast advancement of communication technology and the exchange of information across the internet, utilizing a transfer control protocol as plain text, necessitates the use of encryption to safeguard sensitive data during the transition. The term “crypto” is derived from the Greek word “kryptos”, which means “hidden or secret” [1,2]. Indeed, the use of encryption initially arose as the art of communication itself in 1900 B.C. Furthermore, encryption was designed to change a communication into an unreadable form in order to safeguard it surreptitiously while it was sent from one location to another. After many years of development, cryptography and cybersecurity have evolved in ways of encryption to add aspects to sensitive data such as secrecy, integrity, and authenticity, as is recognized by the CIA [3,4,5]. At present, cryptography includes three aspects, namely, symmetric, asymmetric, and procedural aspects [6,7,8]. From ancient times until 1976, the majority of encryption techniques were symmetric, meaning they utilized the same key for encryption and decryption. Encryption is the process of converting plain text (text that can be read) to unreadable text (written information that cannot be read; ciphertext). Decryption, on the other hand, is a process that turns unreadable text (ciphertext) into readable plain text [9]. Diffie, Hellman, and Merkle introduced asymmetric cryptography in 1976 [10]. In asymmetric cryptography, there are two keys: a public key for encryption and a private key for decryption, according to [11]. The public key may be used by anybody to encrypt data; however, the private key can only be used by the authorized individual to decode the data. Stream and block ciphers are included in symmetric ciphers [12,13]. The stream cipher algorithm encrypts bits one-by-one [14]. It appears to be tiny, speedy, and widespread in embedded devices such as GSM phones. Block ciphers, on the other hand, encrypt the entire block (several bits) which are mostly used and common in internet applications [15]. Encryption is now used in several areas, including biology, math, and physics, to secure private information during network transmission. Text and images are two examples of data types that may be sent via a network. Currently, photos are commonly used; hence, encryption is necessary to make these images safe and confidential [16]. However, one of the most difficult aspects of transferring data through a network is ensuring its security and speed. To address the first issue, a plain picture must be transformed into an encrypted image during the transition to keep the image safe from unwanted users. Picture encryption is a practical method of ensuring image confidentiality, integrity, and availability, since it encrypts digital images before storing them for transmission. The receiver cannot distinguish the original information from the encrypted picture information, and only genuine receivers or users with private cipher keys can decrypt it accurately and successfully. The classical and traditional encryption algorithms cannot achieve this level of security for images, and they also have a low security level when used for image encryption. However, chaos theory, which was used in physics, mathematics, biology, and engineering until the 1980s, and which became the foundation of cryptographic applications, was regarded as a good and, in fact, the best choice to permute each pixel in the image and convert it to a scrambled image that can resist statistical attacks [17]. The development of fifth-generation (5G) wireless networks is gaining traction, with the goal of connecting practically all parts of life via a network with substantially faster speed, very low latency, and pervasive connections. Because of its importance in our lives, the network must protect its users, components, and services [18]. The sensitivity to beginning circumstances, ergodicity, pseudo-randomness, unpredictability, topological transitivity, and parameter changes contribute to the strength of chaos encryption. However, this sort of security takes a long time to process and send pictures over a network [19]. In order to accomplish speed, increase efficiency, provide time gain, and minimize transaction costs, strong hardware architectures are used by leveraging parallelism as an alternate approach to process data. Speed, particularly in information technology, has now become a crucial component affecting many facets of our lives, even in this period dubbed “the age of speed”. As is well-known, the chaos encryption process is the greatest way to ensure security, particularly for images; yet, the sophisticated structure of chaos, as well as the enormous number of iterations and rounds inside its internal states, makes it slower than other encryption algorithms as a result of the preceding and of the critical requirement for an efficient system that can be utilized to encrypt pictures and video. In this research, we suggested a new parallel encryption system based on chaotic maps and the message passing interface to efficiently encrypt pictures and video. This system may be designed to achieve a high level of security while still providing great encryption performance. The remainder of this work is structured as follows. Section 2 examines some of the linked literature. Some parallel programming approaches are shown in Section 3. Section 4 puts the experimental results to the test. Section 5 examines the performance of a novel algorithm. Finally, in Section 6, we will go through the findings.

## 2. Related Work

Encryption algorithms are mathematical procedures that are used to conduct encryption by utilizing two inputs: plain text and a secret key. Effective encryption techniques are required to secure sensitive data and keep them private. Following are several algorithms and publications that are typically related.

### 2.1. Stream Ciphers Algorithms

Many stream cipher algorithms, such as Salsa 20, Rabbit, and HC-128, have been studied in the literature. Salsa20/r is one of Daniel J. Bernstein’s stream encryption algorithms, where r = 8, 12, or 20, which is the number of iterations of the round function. The Salsa algorithm may employ cryptographic keys of 128, 192, and 256 bits, according to [20]. To create 512 internal states, a 64-bit nonce (unique message number), a 64-bit counter, and four 32-bit constants are utilized. A keystream output of 512 bits is considered. Each output block comprises a unique combination of the key, nonce, and counter. Because there is no chaining between blocks, Salsa20/r operates in the same way as a block cipher in counter mode. Salsa20/r generates keystreams with a maximum length of 270 bits. Rabbit, on the other hand, is a stream cipher that was invented in 2004. It is thought to be one of the most effective and sophisticated algorithms. It requires a 64-bit nonce and a 128-bit key as input. After each repetition, it produces a 128-bit result. Its encryption comprises of 513 internal state variables, eight 32-bit counters, and one counter carry bit [21]. It is resistant to a wide range of attacks, including algebraic, correlation, and statistical attacks. Differential fault attacks, on the other hand, were found in 2009. The HC-128 stream cipher algorithm has a 128-bit key and a 128-bit instantiation vector (IV). Its internal state is divided into two tables, each of which includes 512 registers of 32-bit length, and one of which is created by a nonlinear output function; each register is updated in 1024 steps [22]. The HC-265 variant has a 256-bit key length and varies from the HC-128 in that the table size is 1024 32-bit elements rather than 512 32-bit elements.

### 2.2. Related Papers

Abutaha explains and presents a stream cipher method based on chaos theory in his paper [23]. He creates a new chaotic stream generator  [24] with the goal of generating a keystream in order to use the key as the primary component of the encryption technique. His chaos generator structure is made up of an instantiation vector, a key setup, nonvolatile memory, and an internal state that creates the output function as the keystream. Its internal state consists of two recursive cells, each of which includes one of two chaotic maps based on mathematical equations such as the skew tent map (ST) and the piecewise linear chaotic map (PWLC), as well as a linear feedback shift register as a perturbation approach. In addition, he implements this generator in a sequential version and a parallel model based on a shard memory architecture using the Pthread library, but his results show that using a shared parallel model with Pthread is only suitable for large datasets, whereas small datasets are not suitable for Pthread. In his paper  [25], Dariusz describes a parallel technique based on OpenMP that parallelizes a block cipher based on a spatiotemporal chaotic system and a chaotic neural network. He demonstrates that the use of a multicore and multiprocessor may minimize the encryption and decryption times; moreover, his experimental results suggest that time-consuming loops inside the function can be parallelizable. In their paper [26], with a chaos-based encryption scheme based on the logistic map and Fibonacci sequence that is used to convert the logistic map into an integer value, Lui et al. demonstrate the chaos algorithm in both sequential and parallel versions depending on the message passing interface (MPI) using a master/slave communication model through using the master to initialize the parameter, logistic map, and Fibonacci sequence. This methodology is appropriate for the encryption and decryption of sensitive data or multimedia. Ünal Çavuşoğlu et al., in their paper [27], developed a new random number generator (RNG) and a chaos-based parallel encryption algorithm for increasing both the security and speed; they used a parallel data encryption model by dividing an image into the number of used threads and encrypting each part with a separate thread. The partitioning process is computed according to a different number of threads for an image with the size of m columns and m rows, and the rows are divided by the number of threads to obtain n parts of equal size for processing in each thread. The keyspace size of their generated algorithm is $2^{888}$ in the parallel version, instead of $2^{216}$ in the sequential version, according to the use of different initial conditions for each thread. Amany Elrefaey et al. [28] demonstrate a parallel implementation of chaotic-based picture encryption that is dependent on two phases required for chaotic-based image encryption processes: replacement and diffusion. They established a parallelization notion on the Baker method by employing a vectorization form and approach, rather than a loop in the sequential version, by generating a map table just once that maps the new pixel position to the previous pixel position. This table remains the same during all replacement iterations. They accomplished parallelization in the diffusion idea by developing a modified method that divides the picture into n similar-sized divisions and executes the encryption operation on each component individually in parallel, utilizing multiple processor cores. Massod et al.  [13] suggested a new system that is computationally less costly and provides a greater degree of security. The system is built on a shuffling process, using fractals as the key as well as a three-dimensional Lorenz chaotic map. The shuffling procedure applied the confusion attribute and jumbled the pixels of the standard picture. A three-dimensional Lorenz chaotic map is utilized for a diffusion process that distorts all of the image’s pixels.

Massod et al. [29] suggested a lightweight cryptosystem to encrypt medical pictures with enhanced security based on a Henon chaotic map, Brownian motion, and Chen’s chaotic system.

In the next part, we will look at several parallel programming paradigms and multiprocessor architectures.

## 3. Parallel Programming Models and Techniques

In the 1980s, CPU manufacturers such as Intel and AMD enhanced the speed of their computers by expanding the hardware design and increasing the number of transistors for each processor, in order to achieve high performance. This transitional period lasted until the mid-1990s and the beginning of the 2000s. When the clock rate increases, they approach a physical limit created by inhibiting the CPU’s ability to cool itself. Manufacturers boost performance by designing a new CPU architecture with several cores that can accept various instructions and data streams (MIMD). The concept of existing multi-core architecture is based on the idea of utilizing all of these cores by employing parallel programming techniques. The parallel programming paradigms are determined by the hardware architecture, which is either shared memory or distributed memory. Both architectures share the same concept of breaking work down into such units and assigning these units to each core to process individually. All control units (CU) and processing units (PU) are shared with a single memory in a shared memory architecture. In distributed memory, however, each processing unit and control unit has its own local memory that is totally connected via the interconnection network. The need for high speed and high performance of hardware systems, in many aspects, such as artificial intelligence, IoT, real-time applications, video conferencing, and video-on-demand, has led programmers to search for and rely on another way of programming that paved the way to solving problems that concurrent programming encountered consistently. Parallel programming is an alternate option that interfaces directly with hardware to give the required application with great speed and efficiency. Parallel programming appears to be the greatest technique to fully utilize all available resources and processors in order to provide high-speed performance. This style of programming may be used to address mathematical problems in many scientific fields. Parallel programming models and techniques are divided into two categories: shared memory and distributed memory. OpenMP and Pthread are examples of shared memory models, whereas message passing interfaces are examples of distributed memory models. OpenMP is a Fortran and C/C++ application program interface (API). It takes a user program and breaks it down into threads that run on a shared memory basis. OpenMP is a cross-platform program that may operate on any operating system, including UNIX, Linux, and Windows [30]. OpenMP is made up of compiler directives, runtime libraries, and environmental libraries. Pthread, on the other hand, is a library that can be linked with the C/C++ programming languages. It is not a programming language in the same way that Python or Java are. It was sometimes referred to as a POSIX thread  [31]. A standardized extension exists for Posix threads (IEEE Std 1003.1c-1995). Thread management-creating, joining threads, and so on, are some of the numerous processes performed by POSIX threads. Mutexes, condition variables, and thread synchronize through read/write locks and barriers, unlike MPI, which stands for message passing interface  [32]. MPI communicates with other languages such as C/C++ and FORTRAN. MPI is widely regarded as one of the most essential programming languages for utilizing distributed memory architecture. It is almost as though another language has its own data type and function. To communicate between two processors, the first executes a send operation, while the second receives an operation. MPI has several implementations, including MPICH, MPICH2, and OpenMPI. MPI is now binding to other languages by enclosing MPI implementations in other languages such as Java, MATLAB, and Python. In this work, we concentrate on a parallel distributing model based on a message passing interface model, since it has a better capacity to accelerate performance when compared to other parallel models.

## 4. Proposed Parallel Chaos Crypto System

**Figure 1:**
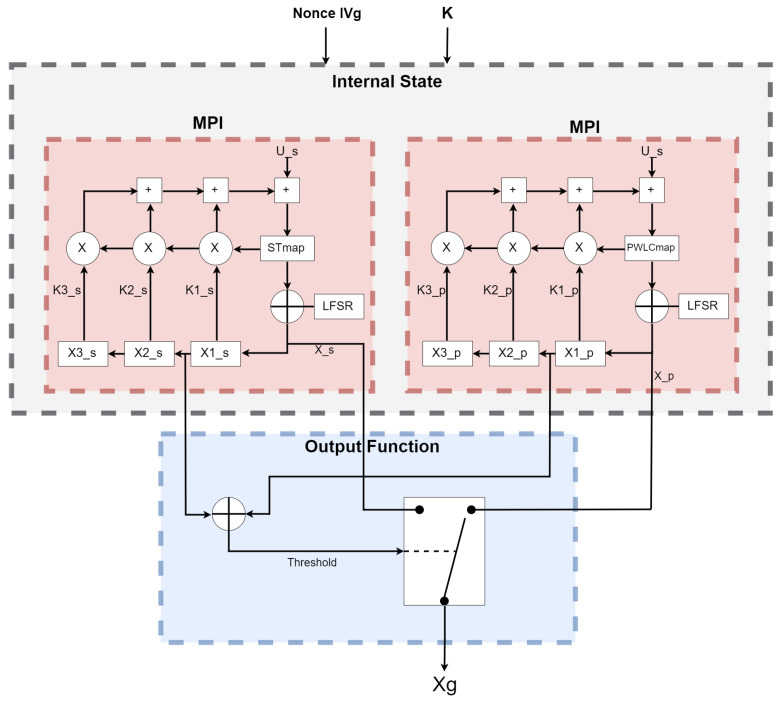
Proposed parallel chaos generator.

**Figure 2:**
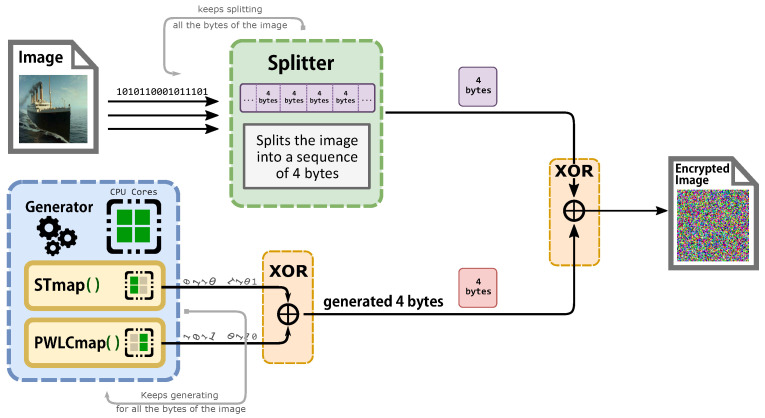
Description of the architecture of the parallel proposed cryptosystem.

The new technique is heavily reliant on two non-recursive filters (skew tent map and PWLC map), which provide randomization and non-periodicity. Because chaos is sensitive to beginning circumstances, increasing the unpredictability will increase the randomness. The beginning circumstances of our algorithm are drastically modified in each iteration. In order to improve a chaotic generator’s efficiency and computing performance, we use MPI for each cell of the non-recursive filter, which allows each cell to function in its own memory space, separate from the others, as seen in Figure 1 and Figure 2. Each process will execute each cell individually, as shown in Algorithm 1 and Figure 2. The system begins by taking a clear image as an input and then converts it into bytes by splitting the image into divisions of four bytes. We add padding if we are unable to split the picture into four-byte chunks. The MPI environment was set up to apply MPI to each recursive cell in order to achieve the required parallel suggested system by distributing the amount of processors and cores evenly to the PWLC and skew tent chaotic maps by calling them concurrently. The result of the two maps is then XOR together to form four bytes. These procedures are continued until the number of created keystream sequences equals the amount of plain image bytes. Finally, encryption is performed for every four produced sequences, with each sequence containing four bytes of keystreams. Those 16 bytes of keystream will XOR with the plain image’s 16 bytes. This technique is continued until the entire image is encrypted. Because the stream cipher is a symmetric encryption technique, decryption follows the same steps as encryption. First, we construct the four bytes’ combination of the encrypted picture. The chaotic generator is then used to produce the same key streams using the same secret key. Finally, the picture is decrypted by XORing the four-byte combinations with the key streams.

The equations of the discrete skew tent and the discrete PWLCM maps are, respectively, given by [24]:

Discrete Skew Tent Map:

(1) $$X_{s}\left[n\right]=\left\{\begin{array}{cc} \left\lceil 2^{N}\times \frac{X{}_{s}\left[n-1\right]}{P1}\right\rceil & \mathrm{if}\;0<X{}_{s}\left[n-1\right]<P1\\ 2^{N}-1 & \mathrm{if}\;X{}_{s}\left[n-1\right]=P1\\ \left\lceil 2^{N}\times \frac{2^{N}-X{}_{s}\left[n-1\right]}{2^{N}-P1}\right\rceil \; & \mathrm{if}\;P1<X{}_{s}\left[n-1\right]<2^{N} \end{array}\right..$$

Discrete PWLCM map:

(2) $$X_{p}\left[n\right]=\left\{\begin{array}{cc} \left\lceil 2^{N}\times \frac{X{}_{p}\left[n-1\right]}{P2}\right\rceil & \mathrm{if}\;0<X{}_{p}\left[n-1\right]\leq P2\\ \left\lceil 2^{N}\times \frac{X{}_{p}\left[n-1\right]-P2}{2^{N-1}-P2}\right\rceil & \mathrm{if}\;P2<X{}_{p}\left[n-1\right]\leq 2^{N-1}\\ \left\lceil 2^{N}\times \frac{2^{N}-P2-X{}_{p}\left[n-1\right]}{2^{N-1}-P2}\right\rceil \; & \mathrm{if}\;2^{N-1}<X{}_{p}\left[n-1\right]\leq 2^{N}-P2\\ \left\lceil 2^{N}\times \frac{2^{N}-X{}_{p}\left[n-1\right]}{P2}\right\rceil & \mathrm{if}\;2^{N}-P2<X{}_{p}\left[n-1\right]\leq 2^{N}-1\\ 2^{N}-1-P2 & otherwise \end{array}\right..$$

The values $X_{s}\left[n\right],X_{p}\left[n\right]$ produced by the recursive cells in the internal state are entered to the output function. Then, the output sequence Xg(n) is obtained by XORing X1_s and X1_p, as clarified in Equation (3):

(3) $$Xg\left(n\right)=X_{s}\left(n\right)⊕X_{p}\left(n\right).$$

**Algorithm 1** PARALLEL IMAGE CHAOS ENCRYPTION ALGORITHM**Input:**  *clear image* **Output:**  *Scrambled Encrypted image*  1:*Convert image into bytes*  2:*Split image into 4 bytes*  3:***Initialize** MPI environment*  4:*MPI_Comm _rank(MPI_COMM_WORLD, &rank)*  5:*MPI_Comm _size(MPI_COMM_WORLD, &size)*  6:**Distribute** *processors equally on the two Cells*  7:*rank ← 0*  8:**if**rank<=(size/2)**then**  9:   *$K->X_{s}=STmap\left(\right)$*10:**else**11:   *$K->X_{p}=PWLCmap\left(\right)$*12:**end if**13:**Finalize** *MPI Environment*14:**Generate** *a sequence of 32 bits $from\;XORing\;K->X_{s}\;and\;K->X_{p}$*15:**Convert** *each 32-bit sequence into 4-byte sequences*16:**XORing** *each 4-byte sequence in the key with the corresponding 4-byte sequence in the image*

**Figure 3:**
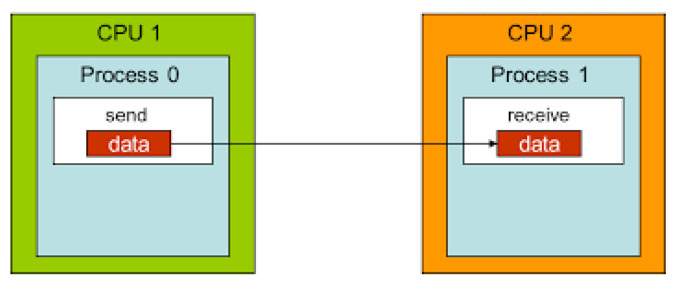
Point-to-point communication.

Based only on the process ID, the MPI system advises each process on which portion of the global problem they should be working on. Large amounts of data, as well as load balancing across processors, need the usage of both point-to-point and group communication. The goal behind employing point-to-point communication is to distribute data over two or more processors in accordance with the computer and workstation specifications. Rather than concentrating all processing power and labor in one location, as indicated in Figure 3, the message contents are transferred to a system-controlled block of memory. Process 0 continues to perform additional tasks without pause. When process 1 is finished, it reads the message from the remote system buffer and puts it in the proper memory address. The suggested approach divides the burden of bits over several cores utilizing MPI, which will raise the average bit rate and lower the number of cycles per byte of data. In general, there are two distinct techniques for using ILP. The first technique depends on hardware to assist, detect, and utilize parallelism dynamically, whereas the second, as in our proposed research, focuses on software technology to find parallelism statically at the compile time. The suggested parallel chaos cryptosystem intends to eliminate reliance on data, names, and controls. We can eliminate data reliance by changing the code and reordering or scheduling the instruction with worries that ordering does not have an effect, resulting in unwanted outcome. We handle this problem by utilizing distinct objects, rather than relying on a single common item, according to the naming dependence.

## 5. Performance Computation of Proposed Parallel Computing

We conduct various tests on two computers to calculate and assess the performance of the proposed parallel cryptosystem based on MPI. The first features an Intel Core i3 (TM) processor running at 1800 GHz and 4 GB of RAM, while the second has an Intel Core i5 (TM) processor running at 2400 GHz and 4 GB of RAM. As a trustworthy Linux version, we operate the parallel cryptosystem on Ubuntu 18.04. In these tests, three metrics are taken into account: the number of cycles required to create one byte (cycle/byte), the generation time in microseconds, and the rate at which bits are transported in megabits per second (Mbit/s), as clarified in Table 1, Table 2, Table 3 and Table 4.

(4) $$NCpB=\frac{CPUSpeed_{\left(Hertz\right)}}{BR_{\left(Mbit/s\right)}}$$

(5) $$BR=\frac{Datasize_{\left(Mbit\right)}}{Gen\_time_{\left(micro_{s}\right)}}$$

(6) $$ET=\frac{Image\_size\left(Mbit\right)}{Encryption\_time_{\left(s\right)}}$$

As seen in Table 1, the number of cycles per byte decreases quicker in MPI as compared to sequential and parallel implementations because MPI uses all parallelisms. Cores can handle both tiny and big amounts of data by distributing and balancing the load evenly among all processors. As indicated in Table 1, NCBP is 44.9 when the data size is modest, such as 2048 bytes in MPI, but 155.9 and 82,950 in sequential and Pthread. When using MPI on huge data sets such as 3,145,278 bytes, the NCPB in MPI is 22.2, whereas the NCPB in seq and parallel is 32.0 and 36.3, respectively. As a consequence of the results, we believe that employing MPI in our cryptosystem is the quickest model in small and large image sizes. In contrast to Table 1, which indicates the number of cycles, Table 2 gives the average time of the chaos generator in both sequential and parallel modes. According to the findings, the average time of a parallel chaos generator based on MPI implementation is less than that of a sequential chaos generator and a parallel chaos generator based on a shared memory architecture (Pthread implementation). That is, MPI uses all of its core parallelism by distributing memory to each core, which works independently of the others. When tiny data is entered into the generator, such as 128 in MPI, the average encryption duration is 11 cycles, whereas the sequential and Pthread versions take 38 and 1389 cycles, respectively. However, when huge amounts of data are entered into the cryptosystem, such as 3,145,728 bytes, the average generation time in MPI is 119,640, but the average generation time in sequential and Pthread is 162,322 and 133,493 microseconds, respectively. As is well-known these days, time is a significant component in determining the efficiency and availability of software programs; so, employing MPI as a parallel model is the ideal option for reducing time and increasing availability by encrypting pictures. As shown in Table 4, which illustrates the result of bit rate, which is the number of bits processed in a unit of time, the bit rate in MPI is 862.32 when the data is 512, while the bit rate in sequential and Pthread implementation is 156.04 and 14.95, respectively. When the size is increased to 3,145,728, the bit rate is 864.79, whereas the bit rate in sequential and Pthread implementation is 620.14 and 754.07, respectively. Why is the bit rate being increased? When compared to other implementations, MPI has a good communication system that allows the processors to communicate and transmit data among themselves, allowing MPI to have the greatest encryption bit rate in both small and huge data.

Nonetheless, when the data is 3,145,278 bytes, the NCpB in MPI is 13.2 and in Pthread is 19.2 and 16.7, respectively as shown in Table 5.

**Figure 4:**
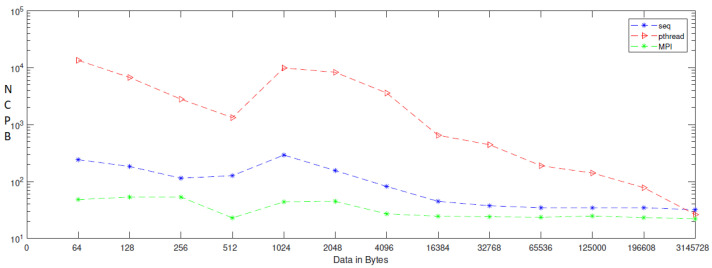
NCPB for sequential, Pthread, and MPI implementation on two cores.

**Figure 5:**
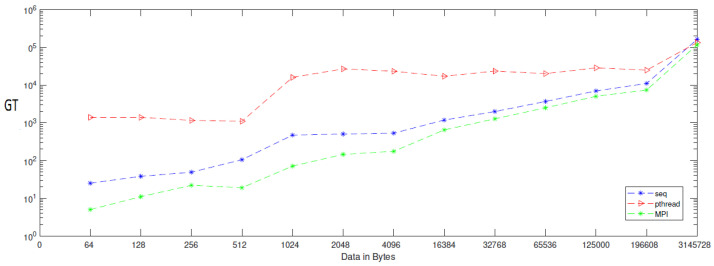
Generation time for sequential, Pthread, and MPI implementation on two cores.

**Figure 6:**
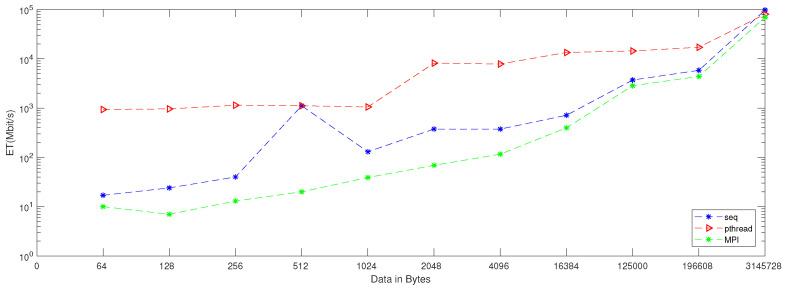
Bit rate for sequential, Pthread, and MPI implementation on two cores.

**Figure 7:**
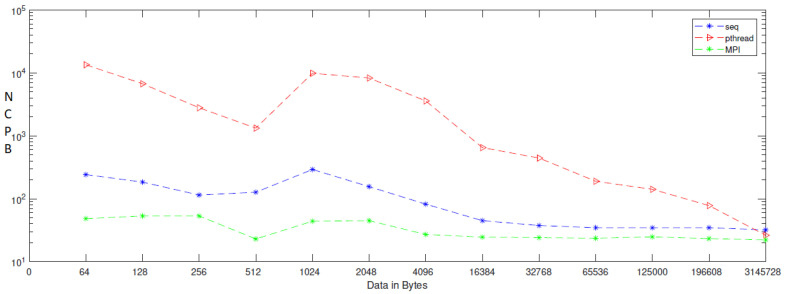
NCpB for sequential, Pthread, and MPI implementation on four cores.

**Figure 8:**
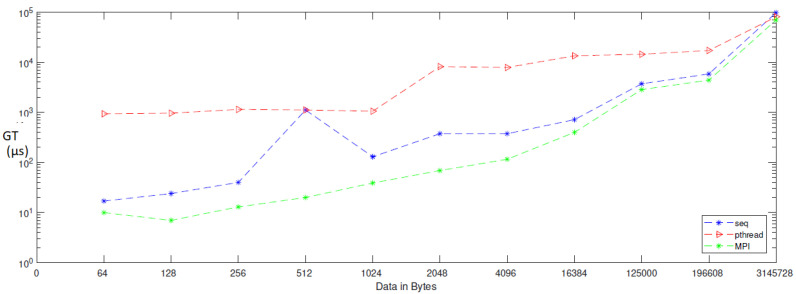
Generation time for sequential, Pthread, and MPI implementation on four cores.

**Figure 9:**
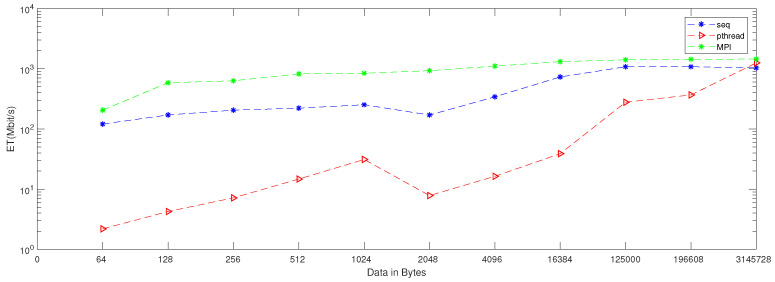
Bit rate for sequential, Pthread, and MPI implementation on four cores.

When applying generation time results on a computer that has four cores, the result, as reported in Table 6, demonstrates that the generation time decreases proportionally with an increasing number of processors. The generation time In MPI is 13, in contrast to the sequential and Pthread implementations, for which the generation times are 40 and 1143, respectively, when the data contains 256 bytes. However, when the data contains 3,145,728 bytes, the generation time is 69,631, but the generation time in sequential and Pthread is 97,584 and 80,568, respectively. As noted from Table 7, which reported the result of the bitrate on a computer that has four cores, that experimental result shows that the bitrate is increasing proportionally with an increasing number of processors on a workstation. The bitrate in MPI is 1103.5 when the data is 4096 bytes, in contrast to the sequential and parallel implementation, whose bit rates are 341.33 and 16.32, respectively. For 196,608-byte data, the bit rate in MPI is 1434.06. However, in Pthread and sequential, the bit rates are 1077.63 and 366.88, respectively. As illustrated in Table 3, this demonstrates a performance comparison result between the stream cipher algorithms and our proposed algorithm. We implement this data by encrypting the Lena image three times, each time with a different size. The result shows that the performance increases in MPI, compared to the others, with an increase in the size of data. Figure 4, Figure 5, Figure 6, Figure 7, Figure 8 and Figure 9 show the curves that depict the NCpB, generation time, and bit rate measurements either in two or four cores for the sequential, Pthread, and MPI implementations.

### 5.1. Speed-Up Calculations

The term ”speed-up” refers to the measuring of parallel code in terms of how much quicker it executes in parallel. Assuming that the time to execute code on one processor is time_seq and the time to run code on N processors is time_parallel, the speed-up is provided in Equation (4) [33]:

(7) $$Speed-Up=\frac{Time\_Seq}{Time\_Parallel}.$$

For assessing bit rate enhancement, divide the bit rate in parallel by the bit_rate in sequential, as shown in Equation (5):

(8) $$BitRate\_Enhancement=\frac{BitRate\_Parallel}{BitRate\_Seq}.$$

The enhancement of a number of cycles per byte can be given as shown in Equation (6):

(9) $$NCpB_{E}nhancement=\frac{NCpB\_Seq}{BitRate\_Parallel}.$$

**Figure 10:**
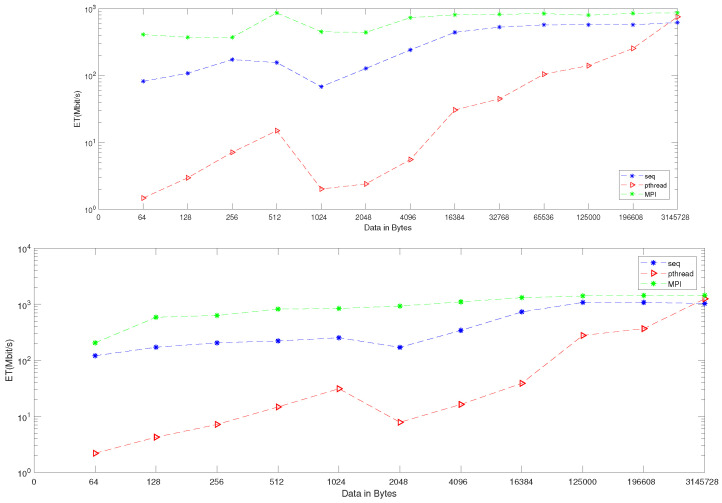
Bit rate enhancement on two and four cores: the upper figure represents two cores, and the lower figure represents the four-core enhancement.

Our results are for the performance shown in Figure 10. The Figures clarified that the performance is better when we applied our algorithm in four cores.

### 5.2. Amdahl’s Law

Amdahl’s law is used to compute an upper bound on the speed-up of an application without actually writing any concurrent code. Each uses the percentage of (proposed) parallel execution time (pctPar), serial execution time (1-pctPar), and the number of threads/cores (p). A simple formulation of Amdahl’s law to estimate the speed-up of a parallel application on p cores is given here [34]:

(10) $$Speed-Up=\frac{1}{1-pctPar+\frac{pctPar}{p}}$$

In our case, we expect 45% of a serial application’s run time could be executed in parallel on four cores; the estimated speed-up, according to Amdahl’s Law, could be as much as (1/(0.55 + 0.45/4) = 1.50943396226). We expect an increase in overhead with an increasing number of cores that will appear, especially, when the number of processors is more than eight.

## 6. Security 

In this section, we will discuss the security analysis of the parallel proposed cryptosystem against cryptanalytic and statistical attacks.

### 6.1. Keyspace

As we know in encryption, the larger keyspace algorithm has a strong ability to resist a brute-force attack, in contrast to algorithms that have a small keyspace and that suffer from a lack of sequence randomness. The proposed cryptosystem has a different keyspace according to the selected delay. The keyspace in delay = 1 is nearly 299 bits; however, in delay = 3, the keyspace reaches 555 bits. This numerous keyspace makes the proposed cryptosystem immune to brute-force attacks. Table 8 lists the size of the keyspace of similar chaotic algorithms. As clarified in Table 8, our algorithm’s keyspace in delay 3 is 555 bits; it seems to be the largest one, compared to the listed algorithms.

### 6.2. Key Security and Sensitivity Attack

To test the key sensitivity of our proposed cryptosystem, we use two important measurements: the number of pixel change rate (NPCR) and the unified average changing intensity (UACI). These show that the proposed cryptosystem is very sensitive to a one-bit change that occurs when we encrypt the “Lena” image more than 100 times with 100 **secret** keys that differ in the LSB bit.

(11) $$NPCR=\frac{1}{L\times C\times P}\sum_{p=1}^{P}\sum_{i=1}^{L}\sum_{j=1}^{C}D\left(i,j,p\right)\times 100\%$$

where:

(12) $$D\left(i,j,p\right)=\{\begin{array}{c} 0,if\;C_{1}\left(i,j,p\right)=C_{2}\left(i,j,p\right)\\ 1,if\;C_{1}\left(i,j,p\right)\neq C_{2}\left(i,j,p\right) \end{array}$$

and the UACI that is used to measure the average intensity difference between the two ciphered images can be defined as follows:

(13) $$UACI=\frac{1}{L\times C\times P\times 255}\sum_{p=1}^{P}\sum_{i=1}^{L}\sum_{j=1}^{C}|C_{1}\left(i,j,p\right)-C_{2}\left(i,j,p\right)|\times 100\%.$$

In the prior Equations (11)–(13), *L*, *C*, and *P* are the height, width, and plane sizes of the image, respectively. *i*, *j*, and *p* are the rows, columns, and plane indexes, respectively. As the results in Table 9 show, the NPCR and UACI values of the proposed cryptosystem are close to the optimal NPCR and UACI values, 99.61 and 33.46, respectively [40].

### 6.3. Information Entropy

The entropy E(X) is statistical measure of uncertainty in information theory [50]. It is defined as follows:

(14) $$E=-\sum_{i=0}^{255}P\left(x_{i}\right)\log_{2}P\left(x_{i}\right)$$

where X is a random variable and $P(x_{i})$ is the probability of the gray value $x_{i}$. Let us consider that there are 256 states of the information source in red, green, and blue colors of the image, with the same probability. We can obtain the ideal E(X) = 8, corresponding to a truly random source. As noted from Table 10, the information entropy of some ciphered images, such as Titanic, Photographer, Manhattan, Cameraman, Lena, and Boat, is closer to 8. The entropy of all ciphers is closer to 8, which proves that the ciphered image is a random dataset of pixels.

Table 11 provides the contrast data with other advanced schemes by comparing the information entropy in our proposed scheme with other advanced algorithms. The result has shown that our proposed scheme has greater superiority in information entropy, compared to the other algorithms.

## 7. Statistical Analysis

### 7.1. NIST Test

**Figure 11:**
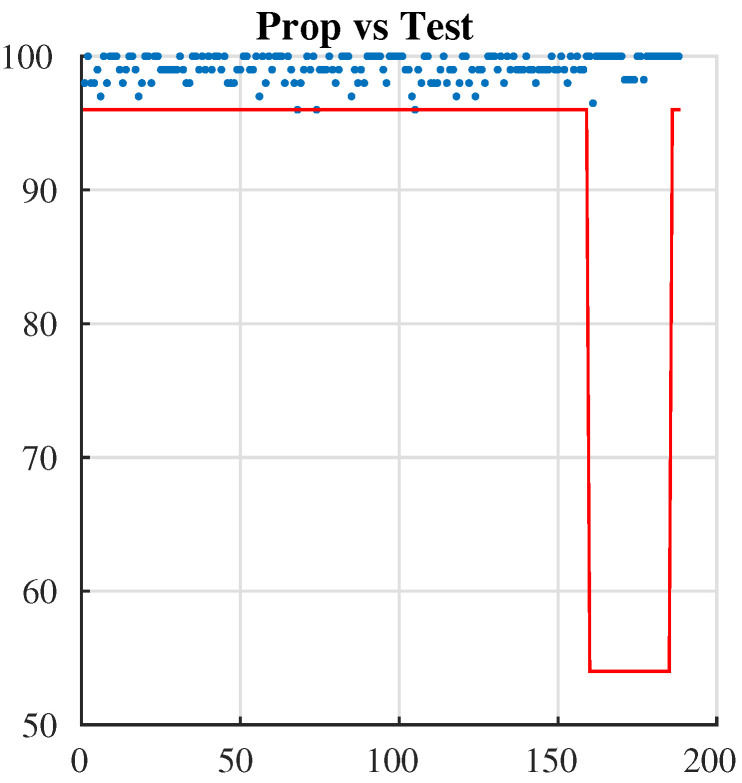
NIST test.

The NIST statistical test suite, which is proposed by the National Institute of Standards and Technology, includes 15 tests that concentrate on different types of non-randomness that could be in a sequence. These tests are cumulative sums (forward), the longest run of ones, cumulative sums (reverse, non-overlapping templates, block-frequency, frequency (mono bit), runs, rank, FFT, overlapping templates, random excursions variant, approximate entropy, serial 1, serial 2, and universal. In order to investigate the randomness of binary data, we apply NIST [51] to many ciphered texts, and the results show that the ciphertext has a high rate of randomness, as shown in Figure 11 and Table 12 [52].

### 7.2. Chi-Square Test and Histogram

**Figure 12:**
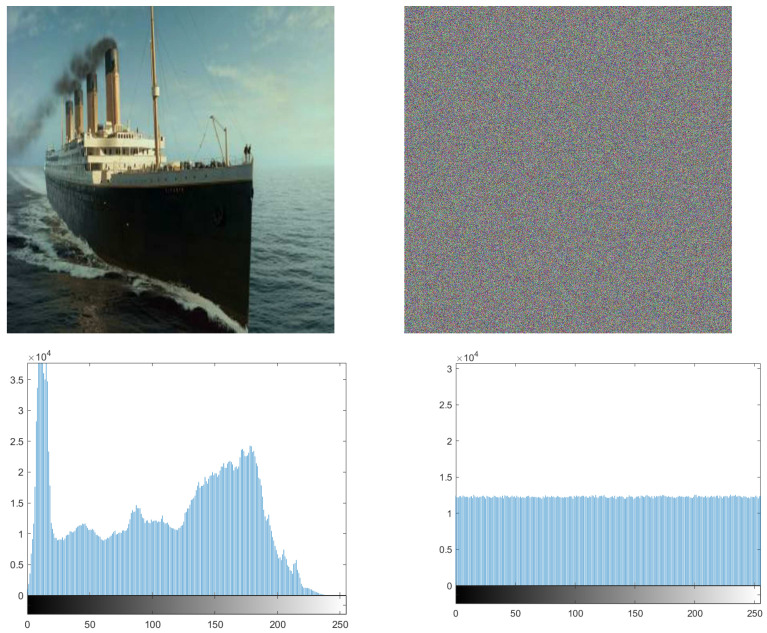
Histogram of the Titanic plain image and its ciphered image.

**Figure 13:**
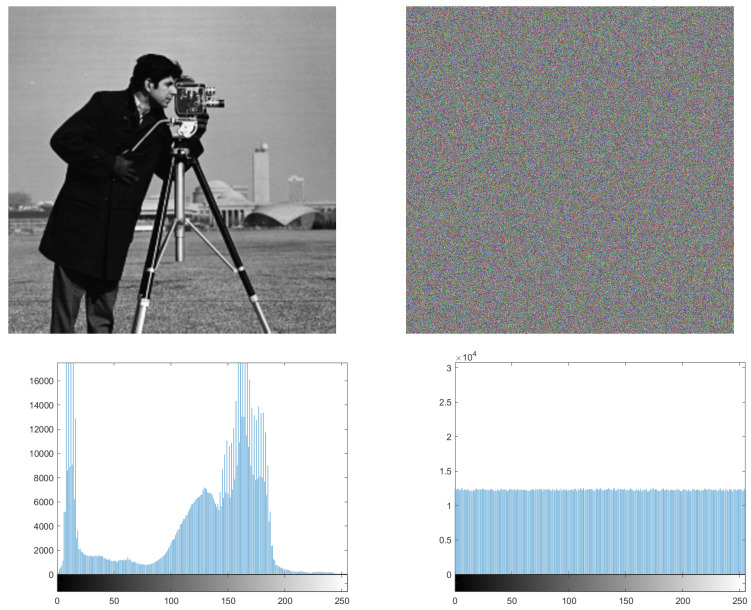
Histogram of the Photographer plain image and its ciphered image.

In order to resist potential and statistical attacks, we apply a histogram, which represents the distribution of pixels in specific images. It is a measure to test the uniformity of a ciphered image. Whenever the histogram in a ciphered image is uniform, the image has more immunity against statistical attacks. For this purpose, we apply the histogram for many pictures and, as seen from Figure 12 and Figure 13, the histogram of the ciphered image is uniform, compared with its plain image.


(15) $$\chi^{2}=\sum_{i=0}^{k-1}\frac{{\left(O_{i}-E_{i}\right)}^{2}}{E_{i}}$$

Equation (10) is used to perform the Chi test, where *K* is the number of levels (here K=256), *O_i_* is the observed occurrence frequency of each color level (0–255) on the histogram of the ciphered image, and *E_i_* is the expected occurrence frequency of the uniform distribution, given here by $E_{i}=L\times C\times P/K$. For a secure cryptosystem, the experimental chi-square value must be less than the theoretical chi-square one, which is 293 in the case of α=0.05 and *K* = 256. In Figure 12 and Figure 13, we give the histograms for the plain/ciphered images for Lena, Boat, Cameraman, and Peppers in size 512×512×3. As we can see, the histogram of the ciphered image seems to be uniform. To assess the uniformity, we performed the chi-square test. The experimental value obtained is less than the theoretical one at 293. This means that the histograms are uniform (see Table 13).

## 8. Correlation Analysis 

The correlation test is an important test, especially in the encryption field, owing to the importance of hiding information from an attacker, and keeping the attacker from deciphering plain-text information from the ciphered text. Thus, in order to achieve security, the correlation of the proposed cryptosystem should be as low as possible. We apply this test by taking 10,000 adjacent pixels from the plain image and the ciphered image, and use them as inputs in Equations (13)–(15).

(16) $$P_{xy}=\frac{Cov(x,y)}{\sqrt{D(x)}\sqrt{D(y)}}$$

(17) $$Cov\left(x,y\right)=\frac{1}{N}\sum_{i=1}^{N}\left(\left[x_{i}-E\left(x\right)\right]\left[y_{i}-E\left(y\right)\right]\right)$$

(18) $$D\left(x\right)=\frac{1}{N}\sum_{i=1}^{N}{\left(x_{i}-E\left(x\right)\right)}^{2}$$

(19) $$E\left(x\right)=\frac{1}{N}\sum_{i=1}^{N}\left(x_{i}\right)$$

**Figure 14:**
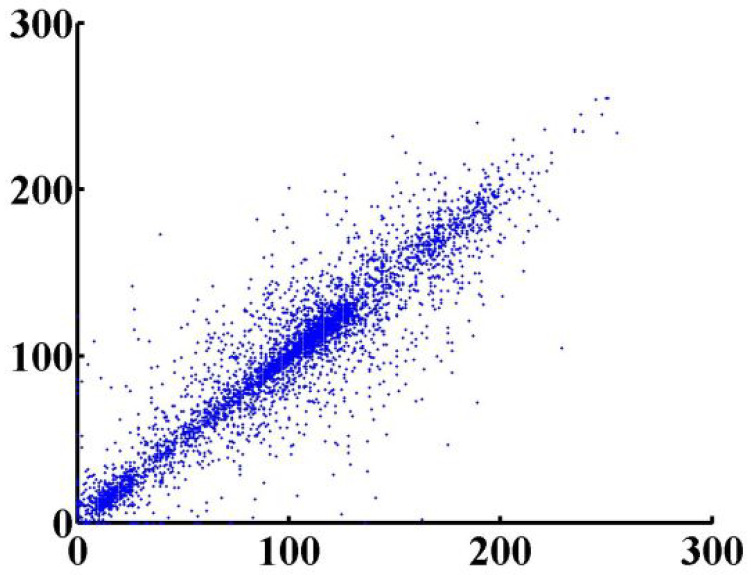
Adjacent pixels for plain image.

**Figure 15:**
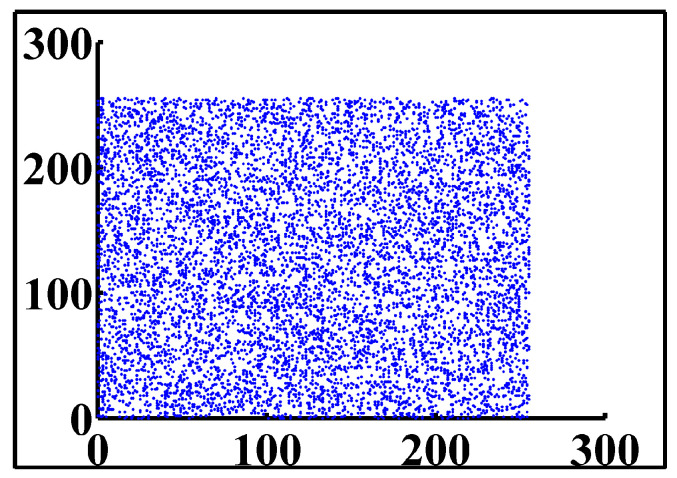
Adjacent pixels for ciphered image.

In the previous equations, $P_{xy}$ is the correlation coefficient of two sequences *x* and *y*. *x_i_* and *y_i_* are the values of *x* and *y*, respectively. For proving that the ciphered image is completely different from the original image, we used a correlation analysis of adjacent pixels for both the ciphered and original image. As the result clarified in Figure 14 shows, adjacent pixels in the plain image are redundant and correlated; however, adjacent pixels in the ciphered image are nearly completely different, and seem to have redundancy and correlation as low as possible (Figure 15). This is more proof that shows that our proposed system has immunity against statistical attacks.

The correlation coefficient values for all previously tested plain/ciphered images are given in Table 14. As we expected, these results conform to those found in the literature.

## 9. Conclusions

We created a parallel chaos generator based on the message passing interface that we consider to be the fastest chaotic stream encryption model due to its generic, random, and non-periodic structure. The proposed system is quicker than previous parallel systems that rely on Pthread or sequential systems. Furthermore, we use a variety of tests on the cryptosystem to assess its efficiency, speed, and security, including the Chi-square and histogram, NIST, correlation analysis, and information entropy tests. All of the tests demonstrated that the proposed cryptosystem is resistant to brute-force and statistical attacks. In addition to the previously mentioned traits and benefits, the results demonstrate that the sequence created by the proposed parallel cryptosystem has a significant degree of unpredictability or uncertainty. Finally, we completed the research objectives by developing a system that combines security and performance. We can adapt our approach to be utilized in video encryption in the future.

## Figures and Tables

**Table 1:** NCpB for sequential, Pthread, and MPI implementation on two cores.

Data	NCpB_Seq	NCpB_Pthread	NCpB_MPI
64	242.1	13,463.2	48.4
128	184.0	6726.8	53.3
256	114.6	2791.9	53.3
512	127.1	1326.9	23.0
1024	292.0	9879.8	44.0
2048	155.9	8295.0	44.9
4096	82.1	3586.4	27.1
16,384	44.9	649.9	24.6
32,768	37.6	443.6	24.1
65,536	34.7	189.8	23.6
125,000	34.6	141.4	24.8
196,608	34.7	78.0	23.3
3,145,728	32.0	26.3	22.2

**Table 2:** Generation time for sequential, Pthread, and MPI implementation on two cores.

Data	Gen_Time_Seq	Gen_Time_Pthread	Gen_Time_MPI
64	25	1390	5
128	38	1389	11
256	49	1153	22
512	105	1096	19
1024	471	15,938	71
2048	503	26,763	145
4096	530	23,142	175
16,384	1180	17,090	646
32,768	1982	23,400	1271
65,536	3665	20,052	2489
125,000	6973	28,523	5008
196,608	10,990	24,748	7391
3,145,728	162,322	133,493	116,401

**Table 3:** Performance results comparison of the stream cipher algorithms.

Algorithm	Image Size	Enc_Time (Ms)	ET (Mbit/s)	NCpB
Rabbit	256×256×3	811.3	1848.8	9.5
	512×512×3	3256	1842.6	9.5
	1024×1024×3	12,950	1853.9	9.5
HC-128	256×256×3	1221	1228.1	14.4
	512×512×3	4895	1225.6	14.4
	1024×1024×3	19,647	1221.5	14.4
Salsa 20/12	256×256×3	836.4	1793.4	9.8
	512×512×3	3389	1770	9.8
	1024×1024×3	13,483	1779.9	9.8
AbuTaha Chaos Stream Cipher(Seq)	256×256×3	5838	1077.63	18.4
	512×512×3	—	—	—
	1024×1024×3	97,584	1031.55	19.2
AbuTaha Chaos Stream Cipher(Pthread)	256×256×3	17,148	366.88	54.1
	512×512×3	—	—	—
	1024×1024×3	80,568	1249.41	16.7
Parallel Proposed Chaos CryptoSystem(MPI)	256×256×3	4387	1434.06	13.8
	512×512×3	—	—	21.2
	1024×1024×3	69,631	1445.65	13.2

**Table 4:** Bit rate for sequential, Pthread, and MPI implementation on two cores.

Data	BR_Seq	BR_Pthread	BR_MPI
64	81.92	1.47	409.60
128	107.79	2.95	372.36
256	173.06	7.10	372.36
512	156.04	14.95	862.32
1024	67.94	2.01	450.70
2048	127.24	2.39	441.38
4096	241.51	5.53	731.43
16,384	442.03	30.52	807.43
32,768	527.95	44.72	823.29
65,536	571.90	104.53	842.11
125,000	573.64	140.24	798.72
196,608	572.45	254.21	851.20
3,145,728	620.14	754.07	864.79

**Table 5:** NCpB for sequential, Pthread, and MPI implementation on four cores.

Data	NCpB_Seq	NCpB_Pthread	NCpB_MPI
64	164.7	9065.9	96.9
128	116.2	4663.7	33.9
256	96.9	2767.7	31.5
512	89.6	1348.7	24.2
1024	78.7	638.0	23.6
2048	116.5	2532.9	21.4
4096	58.1	1215.3	18.0
16,384	27.2	510.9	15.1
125,000	18.4	71.5	14.1
196,608	18.4	54.1	13.8
3,145,728	19.2	16.7	13.2

**Table 6:** Generation time for sequential, Pthread, and MPI implementation on four cores.

Data	Gen_Time_Seq	Gen_Time_Pthread	Gen_Time_MPI
64	17	936	10
128	24	963	7
256	40	1143	13
512	1114	1114	20
1024	130	1054	39
2048	376	8172	69
4096	375	7842	116
16,384	715	13,435	398
125,000	3714	14,416	2844
19,6608	5838	17,148	4387
3,145,728	97,584	80,568	69,631

**Table 7:** Bit rate for sequential, Pthread, and MPI implementation on four cores.

Data	BR_Seq	BR_Pthread	BR_MPI
64	120.47	2.19	204.80
128	170.67	4.25	585.14
256	204.80	7.17	630.15
512	221.41	14.71	819.20
1024	252.06	31.09	840.21
2048	170.21	7.83	927.54
4096	341.33	16.32	1103.45
16,384	729.51	38.82	1310.55
125,000	1077.01	277.47	1406.47
196,608	1077.63	366.88	1434.06
3,145,728	1031.55	1249.41	1445.65

**Table 8:** Keyspace comparison of similar algorithms.

Encryption Algorithm	Keyspace
Proposed Algorithm	$2^{555}$
Wange et al.’s Algorithm [35]	$2^{149}$
Guesmi et al.’s Algorithm [36]	$2^{256}$
Curiac et al.’s Algorithm [37]	$2^{128}$
Curiacet al.’s Algorithm [38]	$2^{357}$
Zhu et al.’s Algorithm [39]	$2^{339}$

**Table 9:** NPCR and UACI measurements.

Cryptosystem	NPCR	UACI
Proposed Cipher Cryptosystem	99.665	33.459
[41]	99.4	32.7
[42]	99.1	32.8
[43]	98.8	31.7
[44]	99.1	32.8
[45]	99.6	33.1
[46]	99.6	33.3
[47]	99.6	33.3
[47]	99.6	33.3
[48]	99.64	33.4
[49]	99.66	33.43

**Table 10:** Information entropy of some ciphered images.

Ciphered Image	Sharukhan	Titanic	Photographer	Manhattan	Cameraman	Boat	Lena
entropy	7.9999	7.9999	7.9999	7.9999	7.9998	7.9997	7.9999

**Table 11:** Information entropy comparison.

Encryption Method	Information Entropy
Proposed Algorithm	7.9999
[41]	7.9973
[42]	7.9975
[43]	7.9977
[44]	7.9973
[45]	7.9982
[46]	7.99
[47]	7.990
[48]	7.908

**Table 12:** Nist test values.

Test	*p*_Value	Proportion
Frequency test	0.494	97.000
Block-frequency test	0.760	100.000
Cumulative-sums test	0.797	97.000
Runs test	0.596	99.000
Longest-run test	0.699	98.000
Rank test	0.029	100.000
FFT test	0.834	98.0000
Nonperiodic-templates	0.479	99.000
Overlapping-templates	0.237	96.000
Universal	0.494	98.000
Approximate entropy	0.740	99.000
Random-excursions	0.223	99.375
Random-excursions-variant	0.428	98.925
Serial test	0.828	99.500
Linear-complexity	0.834	100.000

**Table 13:** Chi-square value of histograms for different ciphered/plain images with different sizes.

Image	Experimental Value	Theoretical Value
Titanic 256×256×3	245.8750	293.247835
Titanic 512×512×3	279.1621	293.247835
Titanic 1024×1024×3	283.5923	293.247835
Photographer 256×256×3	252.1406	293.247835
Photographer 512×512×3	243.8066	293.247835
Photographer 1024×1024×3	251.6162	293.247835
Manhattan 256×256×3	264.6719	293.247835
Manhattan 512×512×3	254.6660	293.247835
Manhattan 1024×1024×3	257.3975	293.247835
Sharukhan 256×256×3	252.9531	293.247835
Sharukhan 512×512×3	228.9316	293.247835
Sharukhan 1024×1024×3	245.7544	293.247835

**Table 14:** Correlation coefficient values for the previous plain/ciphered images.

Plain/Ciphered Image	Horizontal	Vertical	Diagonal
Lena	0.96606/0.035	0.96613/0.026	0.96619/0.027
Boat	0.99605/0.022	0.99703/0.019	0.99671/0.020
Cameraman	0.96618/0.036	0.96771/0.028	0.96767/0.022
Peppers	0.96608/0.019	0.96612/0.031	0.96647/0.011
